# Efficacy of a Commercial PCV2a Vaccine with a Two-Dose Regimen Against PCV2d

**DOI:** 10.3390/vetsci6030061

**Published:** 2019-06-28

**Authors:** Oleksandr Kolyvushko, AGM Rakibuzzaman, Angela Pillatzki, Brett Webb, Sheela Ramamoorthy

**Affiliations:** 1 Department of Microbiological Sciences, North Dakota State University, Fargo, ND 58102, USA; 2 Animal Disease Research and Diagnostic Laboratory, South Dakota State University, Brookings, SD 57006, USA; 3 Veterinary Diagnostic Laboratory, North Dakota State University, Fargo, ND 58102, USA

**Keywords:** vaccines, PCV2d, antibody, PCV2a, ELISA, viremia, pathology

## Abstract

Porcine circovirus type 2, the causative agent of porcine circovirus associated diseases (PCVAD), consists of three major genotypes PCV2a, 2b and 2d. Current commercial vaccines contain the first-identified PCV2a’s capsid protein or whole virions. Outbreaks of PCVAD, caused by the recently identified PCV2d in vaccinated herds have raised concerns regarding the efficacy of current PCV2a vaccines against PCV2d. Thus, the primary objective of this study was to assess the efficacy of a two-dose regimen for the recently reformulated Fostera PCV MetaStim vaccine, to determine if reformulation with the squalene oil adjuvant and two-dose regimen improves the threshold of protection enough to eliminate viremia in a vaccination and challenge model. Two groups of seven pigs each were vaccinated with the commercial vaccine or PBS, and challenged with the PCV2d virus. Strong pre-challenge virus neutralizing responses were detected against all three genotypes. Post-challenge viremia was not completely eliminated as expected but a 2 log_10_ mean reduction in viral load was achieved in vaccinated pigs. Vaccinated pigs had a mean score of 0 for pathological evaluation, while unvaccinated pigs had a score of 6.6. In conclusion, the reformulated Fostera PCV MetaStim PCV2a-based vaccine provided significant heterologous protection and was effective against PCV2d.

## 1. Introduction

Porcine circovirus type 2 (PCV2) is a small DNA virus, which causes post-weaning multi-systemic wasting disease syndrome (PMWS) in weanling piglets. In the years following its discovery, PCV2 was associated with several other clinical manifestations including enteric, respiratory, reproductive, renal and dermatological diseases; now collectively called PCV2 associated diseases (PCVAD) [1,2]. Despite the availability of effective commercial vaccines, PCV2 continues to be a significant economic burden on the swine industry, both as a primary pathogen and due to its effects in exacerbating coinfections [3,4].

The first PCV2 genotype that was distinguished both clinically and serologically from the non-pathogenic variant called PCV1, was designated as PCV2a. Early commercial vaccines against PCV2a were introduced in the U.S. market in 2006. They were highly effective in preventing clinical signs and thus, curtailing economic losses [5,6]. Although a DNA virus, the rates of mutation for PCV2 are comparable to that of RNA viruses. High prevalence rates, co-infections of the same host with multiple genotypes of the virus, recombination and mutation events are factors that facilitate the emergence of new PCV2 variants in the field [7,8]. Following the introduction of commercial vaccines, the observation that PCV2 outbreaks were occurring in some vaccinated herds led to the identification of a new genotype, designated PCV2b. While PCV2a was predominant prior to 2006, PCV2b rapidly became the predominant genotype thereafter. Several experimental and field studies demonstrated that the PCV2a vaccines were effective in preventing clinical signs due to PCV2b [9,10] but did not eliminate viral shedding or viremia. More recently, in 2011, PCV2 once again evolved into a new genotype called PCV2d, which has displaced PCV2b as the predominant, worldwide genotype [11,12]. Two other genotypes, PCV2c and 2e are reported in literature, but not associated with significant clinical disease [4].

Commercial PCV2 vaccine formulations may contain whole, inactivated PCV2a virus (Circovac, Merial, Duluth, GA, USA), subunit proteins of the PCV2a capsid (Circoflex, Boehringer Ingelheim Vetmedica, Athens, GA, USA, Circumvent PCV2 Intervet/Schering-Plough Animal Health, De Soto, KS, USA, Porcillis PCV, Schering-Plough-Merck, De Soto, KS, USA or inactivated, chimeric PCV1-2a virus (Fostera™ PCV, Zoetis Animal Health, Parsippany, NJ, USA). The vaccines are typically administered to piglets at about three weeks of age. Sows can be vaccinated with two doses, prior to farrowing. Although a majority of the vaccines only require a single dose, Fostera™ PCV and Circumvent PCV2 can be administered in two doses, given two weeks apart. The two-dose regimen is recommended in the presence of maternal antibodies [6,13].

Despite the periodical emergence of new PCV2 genotypes, commercial vaccines against PCV2 are still directed against the earliest detected genotype, PCV2a. While PCV2a vaccines elicit cross-reactive, cross-neutralizing antibodies and are effective in preventing clinical signs due to the heterologous strains, they do not completely prevent viral replication or vertical and horizontal transmission [14]. The emergence, rapid spread and genotype displacement with new PCV2 variants every few years raises concerns about whether the threshold of protection afforded by current vaccines is adequate [15]. In the current context of PCV2d gaining succession as the predominating genotype globally [16], evaluating the efficacy of current PCV2a vaccines against PCV2d becomes critical.

Other studies conducted in the U.S. and Asian countries to evaluate commercial PCV2a vaccines against PCV2d showed that the PCV2a vaccines reduce PCV2d viremia and pathological lesions in challenge models [17,18]. Most of these studies employed a one-dose regimen. Since PCV2a vaccines are already effective in providing protection against clinical outcomes against heterologous strains, further increasing the threshold of protection could reduce transmission and viral evolution. For example, achieving elimination of viremia could have significant implications for curtailing transmission and viral evolution in the field.

The inactivated, chimeric PCV1-2a vaccine (Fostera™ PCV, Zoetis Animal Health) is composed of the PCV2a capsid protein expressed from the backbone of the non-pathogenic PCV1 virus [19]. Following reports of incomplete inactivation leading to recombination with field strains [20], this vaccine was withdrawn from the market but later reformulated and re-introduced in 2011. In 2015, a squalene oil adjuvant called MetaStim was added to the formulation (Fostera^®^ PCV MetaStim^®^, Zoetis, Inc., Parsippany, NJ, USA). The new product has not been tested against PCV2d before and is optimized for either a one or two-dose regimen. A majority of weanling piglets in the field have circulating maternal antibodies at three weeks of age, when the primary vaccine is administered. Hence, the objective of this study was to test the efficacy of the two-dose regimen of the Fostera PCV vaccine containing the oil adjuvant against PCV2d challenge in a weanling pig model.

## 2. Materials and Methods

### 2.1. Cells and Viruses

The PCV free porcine kidney cell line, PK-15N (005-TDV, National Veterinary Services Laboratory, Ames, IA, USA) was used for virus culture. The PCV2d strain described in GenBank accession number JX535296.1 [12] was used to prepare the challenge virus. For the virus neutralization assay, PCV2a (AF264042.1) and PCV2b (EU340258.1) were used.

### 2.2. Preparation of the PCV2 Virus Cultures

A cloned copy of the PCV2d genome (JX535296.1) [12] housed in the TA cloning vector pCR2.1 (Thermo Fisher Scientific, Waltham, MA, USA) was provided by Dr. X.J. Meng, Virginia Tech and Dr. Tanja Opriessnig, University of Edinburgh. The genome copy was dimerized as previously described [21], such that two tandem copies of the genome were inserted into the pBlueScript II SK (+) (Stratagene, La Jolla, CA, USA) vector. Dimerized infectious clones of PCV2a [21] and PCV2b [22] were previously available. The dimerized infectious clones were transfected into PK-15 cells to rescue recombinant, pure cultures of each PCV2 virus, as previously described [23]. The virus culture obtained was titrated in PK-15 cells. Viral replication in infected wells was scored after staining with a PCV2 specific monoclonal antibody (Rural Technologies, Brookings, SD, USA) to obtain the TCID_50_ value of the culture, as previously described [23].

### 2.3. Vaccination and Challenge of Pigs

All animal experimentation was carried out in compliance with the Institutional Animal Care and Use Committee (IACUC) and Institutional Biosafety Committee (IBC) regulations of N. Dakota (NDSU) and S. Dakota State Universities (SDSU) (IACUC protocol 15-003A). Fourteen 3–4 weeks old piglets, which had detectable antibody titers but were PCR negative (serum was tested) for PCV2 were randomly divided into two groups of seven pigs each. The piglets were PCR negative for other major swine pathogens including PRRSV, swine influenza virus and Mycoplasma spp. One group (*n* = 7) was vaccinated with the commercial PCV2 vaccine Fostera^®^ PCV MetaStim^®^ (Zoetis, Inc.) on days post vaccination (DPV) 0 and 14, following the label instructions for the two-dose regimen. The second group (*n* = 7) served as unvaccinated controls, and was administered equivalent volumes of PBS. In order to assess antibody responses to vaccination by Enzyme Linked Immunosorbent Assay (ELISA) and post-challenge viral loads by qPCR, serum samples were collected on days post vaccination (DPV) 0, 11, 18, 25, 32, 42 and 53. All animals were challenged on DPV 32 with 4 mL of 10^5^ TCID_50_/mL of PCV2d virus (2 mL intranasal and 2 mL intramuscular). Temperatures were recorded and animals were observed for clinical signs of PCVAD during the post-challenge period. Body condition was assessed using a standard scoring system [24]. The animals were humanely euthanized at 21 days after challenge. Weights were taken before challenge and necropsy to assess differences in weight gain. The development of gross lesions in major organs due to the PCV2d challenge was assessed at necropsy. Lung, tonsils, liver, kidney, spleen, tracheobronchial lymph node, mesenteric lymph node and thymus were collected for microscopic assessment.

### 2.4. Antibody Responses to Vaccination

**Figure 1:**
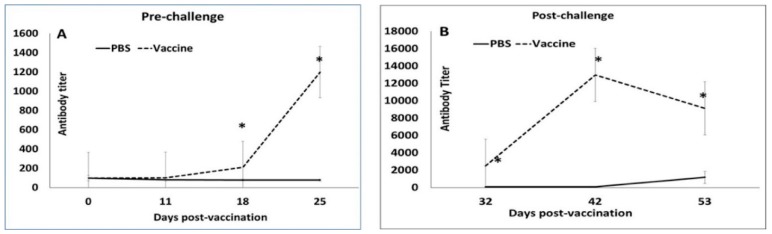
Antibody responses to vaccination: Mean post-vaccination porcine circovirus 2-specific IgG titers by ELISA. X-axis—days post-vaccination, Y-axis—antibody titer calculated as the last dilution showing an optical density higher than the positive cut off. Solid line—unvaccinated group, dashed line—vaccinated group, * *p* < 0.05.

Serum samples, collected as described above, were tested using a commercial PCV2 ELISA kit (Ingezim Circovirus IgG kit, Ingenasa, Madrid, Spain), following the manufacturer’s instructions and the standard operating procedures of the Iowa State University Veterinary Diagnostic laboratory. The test provides a quantitative output measured as an antibody titer, which is the last dilution showing an optical density (OD) (at 550 nm) higher than the positive cut off as well as qualitative measurement of the signal to positive control ratio. The antibody titer was used for further analysis in this study (Figure 1).

### 2.5. Virus Neutralization Assay

**Figure 2:**
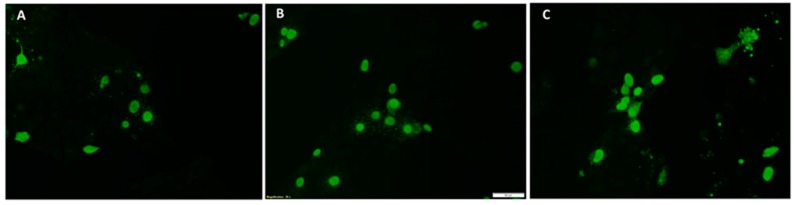
Rescue of recombinant virus culture from transfected PK-15 cells. Representative images of an immunofluorescent assay (IFA) of PK-15 cells transfected with the respective PCV2 infectious clones. Green nuclear florescence typical of PCV2 replication was detected using a PCV2-specific monoclonal antibody. (**A**) PCV2a; (**B**) PCV2b and (**C**) PCV2c.

**Figure 3:**
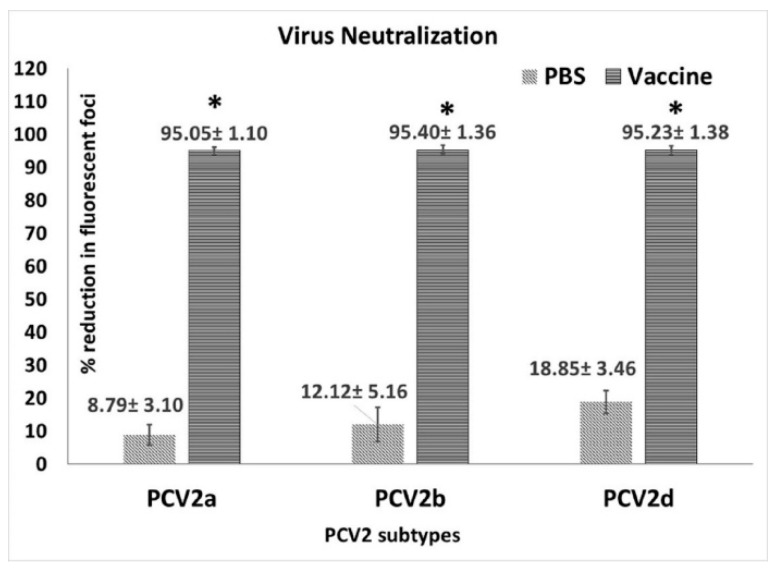
Virus neutralization assay: Virus neutralizing antibodies measured by a fluorescent focus neutralization assay using days post vaccination 35 pre-challenge sera. X-axis—virus genotype used in the assay, light slanted lines—unvaccinated group, dark horizontal lines—vaccinated group Y-axis—mean % reduction in fluorescent foci compared to the untreated virus culture. * *p* < 0.05.

A rapid fluorescent focus neutralization (FFN) assay was performed to measure virus neutralizing antibody responses essentially as described before [25,26] with a few modifications. The PCV2a, b and d virus stocks (Figure 2) were adjusted to 80–100 fluorescent focus units (FFU)/100 uL in DMEM. Test sera were heat inactivated at 56 °C for 30 min and diluted to 1:128 in Dulbecco’s modified eagle’s medium (DMEM, Corning Cellgro, Tewksbury, MA, USA). Equal volumes of the diluted serum and virus were mixed in U bottom plates and incubated for 1 h at 37 °C and then added to a 96 well culture plate containing 30%–50% semi-confluent PK-15 cells. Virus only control samples with no serum were included in each plate. Each sample was assessed by four replicate values. The fluorescent foci were visualized by an indirect immunofluorescence assay performed essentially as described before [23]. Briefly, fixed cells were incubated with a 1:1000 dilution of a PCV2-specific monoclonal antibody (Rural Technologies, Brookings, SD, USA) at 37 °C for 2 h, followed by FITC labeled goat anti-mouse IgG for 45 min. Plates were read under a fluorescent microscope in a blinded fashion. Results were expressed as the % of reduction in the number of foci for respective samples compared to the virus only control (Figure 3).

### 2.6. Quantification of Viral Loads by qPCR

**Figure 4:**
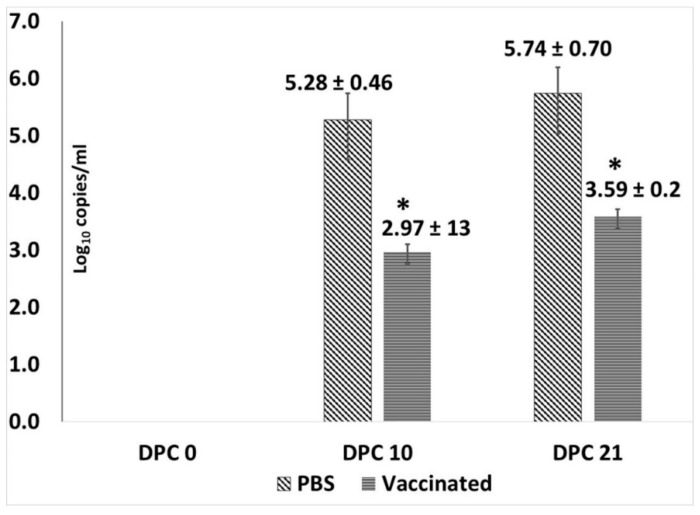
Post-challenge viral loads in pigs: Mean log_10_ genome copies/mL of serum as measured by a PCV2-specific qPCR using DPC 0, 10 and 21 sera. X-axis—days post-challenge, Y-axis—log_10_ genome copies/mL, light slanted lines—unvaccinated group, dark horizontal lines—vaccinated group, * *p* < 0.05.

Total genomic DNA was extracted from test sera with the QIA Amp DNA mini kit (Qiagen, Valencia, CA, USA) and eluted into 100 μL of nuclease free H_2_O. The qPCR reaction to quantify viral copy numbers was performed essentially as described before [27], except that only 1 uL of DNA was used in the reaction. A primer concentration of 0.4 μM, probe concentration of 0.1 μM, and Tm of 65 °C was used with the QuantiFast Probe PCR Kit (Qiagen) and CFX96 Touch qPCR thermocycler (Bio-Rad, Hercules, CA, USA). The lowest limit of detection of the assay was 1 copy/μL (Figure 4).

### 2.7. Pathological Evaluation

The presence of lesions induced by the challenge virus was assessed essentially as described before [22] with minor modifications. For the assessment of gross lesions, the total percentage of lung parenchyma affected was scored in a blinded fashion from 1%–100%. Enlargement of superficial inguinal lymph nodes were scored from 0–3, with 0 being normal, 1 being twice the normal size, 2 being three times the normal size and 3 being four times the normal size. Lung, tonsils, liver, kidney, spleen, tracheobronchial lymph node, mesenteric lymph node and thymus were fixed in 10% buffered formalin for 48 h and transferred to 70% ethanol before being sectioned and routinely processed into 5 μm thick sections for examination. Immunohistochemical staining was performed at the South Dakota State University Veterinary Diagnostic Laboratory, following standard protocols using a PCV2-specific monoclonal antibody. Immunoreactivity was assessed in a blinded fashion by a board-certified pathologist, on a scale of 1–4; where 1 = single follicle or focus staining, 2 = rare to scattered staining, 3 = moderate staining and 4 = strong widespread staining (Table 1).

### 2.8. Statistical Analysis

The qPCR data was transformed to a log10 basis before analysis. The Student’s *t*-test was used to analyze ELISA and qPCR data. Pathological lesion scores and virus neutralization titers were analyzed by the Mann Whitney U test. The level of significance was set at *p* < 0.05 for all analyses. Analyses were performed using the Minitab software (Version 18, Minitab, State College, PA, USA) or Microsoft Excel.

## 3. Results

### 3.1. Booster Vaccination Induces Strong Antibody Responses

All the 14 pigs selected for the study had a detectable but low PCV2 antibody titer (mean value of 104.64) on day 0 (Figure 1A), indicating the presence of low-level maternal antibodies. Following the primary vaccination, antibody responses remained low in vaccinated pigs. However, after the booster, titers increased significantly between DPV 18 and DPV 32 in vaccinated animals, while they continued to remain low in unvaccinated pigs (Figure 1A). A very strong anamnestic response was noted in vaccinated pigs after challenge (Figure 1B). The antibody titers for vaccinated pigs were significantly higher than the unvaccinated group at all the time points after DPV 18 (Figure 1).

### 3.2. Vaccination Induces Strong Virus Neutralizing Antibody Responses

When neutralization responses against homologous and heterologous PCV2 genotypes were assessed using the pre-challenge sera collected at DPV 35, strong responses were detected against all three genotypes (PCV2a, 2b and 2d) (Figure 2) tested. The titers of vaccinated pigs were significantly different from the unvaccinated pigs but there were no significant differences between virus neutralization responses to the different genotypes (Figure 3).

### 3.3. Vaccination Significantly Reduces Challenge Viral Replication

Assessment of protection against PCV2d viremia by qPCR, showed that the PCV2d virus replicated well in the unvaccinated controls with log viral genome copies increasing between day 10 and day 21 post challenge (DPC 21; Figure 4). Only two of the seven vaccinated animals were positive by qPCR on both DPC 10 and DPC 21. There was a significant difference in mean log copy numbers between the vaccinated and unvaccinated pigs on both DPC10 and DPC 21, respectively. Virus was not detected in the serum of any pigs prior to the challenge or on the day of challenge (Figure 4).

### 3.4. Clinical Observations

**Figure 5:**
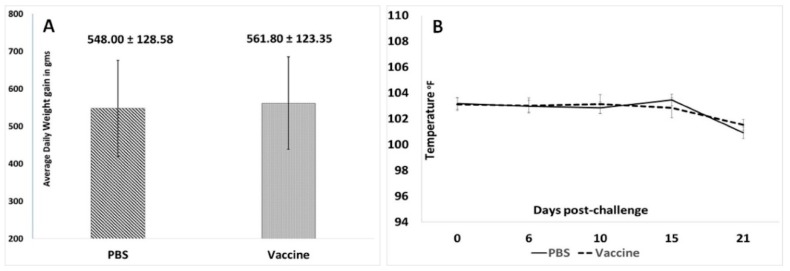
Post-challenge clinical observations: (**A**) Mean average daily weight gain during the 21-day post challenge observation period. X-axis—groups, Y-axis—average daily weight gain in grams. (**B**) Mean body temperatures during the 21-day post challenge observation period. X-axis—days post-challenge, Y-axis—temperature in °F, solid line—unvaccinated group, dashed line—vaccinated group. No significant differences were found between the groups.

In this study, overt clinical signs associated with PCVAD were not observed in either study group. While the average daily weight gain of the vaccinated pigs trended higher than that of the unvaccinated pigs, the difference was not statistically significant (Figure 5A). Similarly, changes in body temperatures (Figure 5B) did not vary significantly between the two experimental groups.

### 3.5. Vaccination Prevents the Development of Lesions

During necropsy, two pigs from the unvaccinated group were found to have a moderate enlargement of the mesenteric lymph nodes and patchy interstitial pneumonia. No other significant gross changes were noted in the major organs of the other pigs. Scattered to strong positive immunoreactivity, indicating the presence of PCV2 antigen, was detected by immunohistochemistry (IHC) in the mesenteric lymph nodes and ileum of 6/7 unvaccinated pigs. Moderate staining was found in the tonsils of all unvaccinated pigs and scattered staining in the tracheal lymph nodes and spleen of 3/7 and 2/7 unvaccinated pigs respectively. No PCV2 antigen was detected in the lung, heart or liver of unvaccinated pigs by IHC. No gross or microscopic lesions and no IHC staining were detected in any of the tissues or organs of any of the vaccinated pigs. The combined pathology and IHC staining scores were significantly different between the vaccinated and unvaccinated pigs (Table 1).

## 4. Discussion

Studies targeted at evaluating the efficacy of currently available commercial PCV2a vaccines against PCV2d have consistently demonstrated a reduction in viremia and protection against PCV2d [11,17,28,29]. These findings are similar to earlier studies evaluating the efficacy of PCV2a vaccines against PCV2b, when PCV2b emerged [6,10]. However, the emergence and rapid spread of the third new PCV2 genotype, PCV2d [16], within the last decade supports the premise that the threshold of protection offered by current PCV2 vaccines has room for improvement. Strategies such as the addition of appropriate adjuvants or providing booster vaccinations, as is possible with Fostera^®^ PCV MetaStim^®^, could further enhance heterologous vaccine mediated immunity to reduce or eliminate viremia.

Two other studies have evaluated the single dose regimen of the non-adjuvanted Fostera vaccine against PCV2b in a field setting [30] and PCV2d under experimental conditions [29]. Sero-positive, viremic piglets were used in both studies to simulate field conditions. While direct comparisons are not possible due to experimental differences between studies, similar to Opriessnig et al. [29] significant differences between vaccinated and non-vaccinated groups in clinical parameters such as weight gain or temperature were not seen in our study, perhaps because of the small sample size and short duration of the observation period.

The timing of PCV2 vaccines, the number of doses and vaccinating in the presence of maternal antibodies are topics of long-standing debate. Sero-conversion is generally delayed in piglets with high maternal antibodies compared to piglets with low maternal antibodies [31,32,33] and two dose vaccine regimens elicit stronger pre-challenge antibody responses than one-dose regimens [29,34]. Consistent with these findings, post-vaccination antibody responses in this study, remained low until administration of the booster, after which, titers increased significantly. However, priming of the immune response appeared to be very effective as the anamnestic response to challenge was several magnitudes higher than the pre-challenge response (Figure 1). In one study, the measurement of cross-neutralizing antibody responses elicited by PCV2a vaccines showed that the response to PCV2a is stronger than the response to the heterologous PCV2b or PCV2d genotypes [17]. In this study, virus neutralization responses as measured by the fluorescent focus neutralization assay, did not differ between genotypes with equally strong responses detected for all three genotypes indicating that the two-dose regimen is effective in eliciting cross-protective neutralizing antibody responses genotype. While it is generally accepted that one-dose regimens provide sufficient protection against PCV2 replication in both homologous and heterologous challenge models [35,36] data from studies employing a two-dose regimen show that the two-dose vaccination is more effective at reducing viremia than a one-dose regimen [29,34] but does not eliminate viremia. Similar to these studies, PCV2d viremia was reduced by approximately two logs in vaccinated animals in this study and was detected in only 2/7 vaccinated pigs (Figure 4). In other studies evaluating the efficacy of current commercial vaccines against PCV2d using a single dose regimen [17,29,30,36], PCV2 antigen was detected in tissues and lymphoid organs. While direct comparison is not possible as a single dose group was not included in this study, PCV2 antigen was undetectable in multiple tissues or lymphoid organs by immuno-histochemistry in this study. The absence of staining for viral antigen in the tissues of the two pigs with low levels of viremia is consistent with the observations that current vaccines protect against clinical manifestation but not viremia or transmission. While immuno-histochemistry assays are less sensitive than PCR based assays, it is possible that effective cell mediated immunity elicited by vaccination was responsible for viral clearance in the tissues but that antibodies were less effective at clearing non cell associated virus.

Therefore, under the experimental conditions used in this study, a two-dose regimen of the PCV2a- based Fostera^®^ PCV MetaStim^®^ was effective in eliciting strong PCV2-specific anamnestic antibody responses, reducing PCV2d viremia and protecting vaccinated pigs against PCV2d challenge induced pathology, supporting a continued role for current vaccines in controlling PCVAD in the field. However, given the success of current vaccines, strategies that can further improve cross-protective immunity may help in the eventual eradication of PCV2 in production herds [6].

## Figures and Tables

**Table 1:** Lesion Scores.

Group	Overall Lesion Score ^%^ (No. of Positive Animals/Total Animals)	Overall IHC Score ^&^ (No. of Positive Animals/Total Animals)	Total Mean Pathology Score (No. of Positive Animals/Total Animals) ^#^
PBS	3.43 ± 8.22 (2/7)	9.14 ± 3.98 (7/7)	27.44 ± 13.04 (7/7)
Vaccinated	0 (0/7)	0 ^a^ (0/7)	0 ^a^ (0/7)

%—Mean lesion scores for major organs. Lymph node enlargement scored as 0 = normal to 3 = severe, pneumonia scored from 0%–100% to represent the % area of affected lung. &—Mean immunohistochemistry (IHC) for the lung, tonsils, liver, kidney, spleen, tracheobronchial lymph node, mesenteric lymph node and thymus scored on a scale of 1–4; where 1 = single follicle or focus staining 2 = rare to scattered staining, 3 = moderate staining and 4 = strong widespread staining. #—Sum of the lesion scores and IHC scores divided by the number of animals. a—significantly different (*p <* 0.05) from the PBS group by the Mann Whitney U test.
